# Wind- and rain-driven macroplastic mobilization and transport on land

**DOI:** 10.1038/s41598-024-53971-8

**Published:** 2024-02-16

**Authors:** Yvette A. M. Mellink, Tim H. M. van Emmerik, Thomas Mani

**Affiliations:** 1 Hydrology and Environmental Hydraulics Group, Wageningen University and Research, Wageningen, The Netherlands; 2 The Ocean Cleanup, Rotterdam, The Netherlands

**Keywords:** Environmental impact, Hydrology

## Abstract

Wind and rain are considered main drivers for mobilization and transport of macroplastics on land, yet there is a lack of empirical data that quantifies this. We present lab experiment results on land-based macroplastic mobilization and transport. We placed four types of macroplastics on terrains with varying surface roughness and slope angles, and exposed them to changing wind speeds and rain intensities. In general, we find that the mobilization probability and transport velocity of macroplastics strongly depend on the combination of the terrain characteristics and material properties. At Beaufort 3, 100% of the plastic bags were mobilized, whereas for the other plastic types less than 50% were mobilized. We found 1.4 (grass) to 5 times (paved surface) higher mobilization probabilities on land than assumed by existing plastic transport models. Macroplastic transport velocities were positively correlated with wind speed, but not with rain intensity. This suggests that macroplastics are not transported on land by rain unless surface runoff develops that can bring the macroplastics afloat. Macroplastic transport velocities were, driven by wind, 1.9 and, driven by rain, 4.9 times faster on paved surfaces than on grass. This study enhances our understanding of land-based macroplastic transport and provides an empirical basis for models.

## Introduction

Plastic pollution of the terrestrial and aquatic environment is a high priority for many governments worldwide as it exerts adverse effects on ecosystems and human health^1,2^. The majority of mismanaged plastic waste that enters the natural environment has a land-based source^3^. Only a small fraction is emitted into the ocean, and most is hypothesized to accumulate on land and within rivers^4,5^. To forecast where plastic waste transports towards and where it accumulates, a better fundamental understanding of the mobilization and transport dynamics of plastic waste on land is required. Such insights are essential for the development of monitoring strategies and effective prevention measures that aim to reduce plastic pollution.

Several studies have hypothesized that the mobilization and transport of macroplastic (> 0.5 cm) on land is largely controlled by wind and rain. Wind was found to be one of the major removal pathways of plastic waste from open landfills^6–8^. Significant positive correlations have been found between wind force and macroplastic abundance on riverbanks^9^ and downwind beaches^10–13^. Additionally, extreme wind storms, e.g., hurricanes, tornados, typhoons, can (re)mobilize and transport macroplastic litter on land^14–16^. In contrast to wind-driven macroplastic transport, where the wind force is directly exerted on plastic items, rain-induced macroplastic mobilization and transport involves an intermediary step: surface runoff. Surface runoff develops when rainwater does not fully infiltrate into the ground. Surface runoff has been shown to be a carrier of microplastics (< 0.5 cm)^17–19^. However, the extent to which surface runoff can carry macroplastic on land is largely understudied. Studies such as the ones by Weideman et al.^20^ and Treilles et al.^21,22^ reported high macroplastic concentrations in stormwater outlets after a rainstorm. Other studies report that during rainier seasons, more plastic debris is flushed from land to the sea^23,24^. Modelling studies on macroplastic transport and emission to rivers and seas adopt positive relationships to varying degrees between wind speed and macroplastic transport, and between rain intensity (or cumulative annual rainfall) and macroplastic transport^25–28^. However, no study to date has empirically quantified and validated such relationships.

To fill this gap, we performed a laboratory experiment using an artificial hillslope to empirically quantify wind- and rain-driven macroplastic mobilization and transport on land. A total of four variables were considered: (i) the driving force magnitude (wind and rain), (ii) the type of macroplastic (bag, wrapper, bottle, and cup; see Fig. S1), (iii) the terrain roughness (paved and grass), and (iv) the terrain slope (0°, 10° and 20°). The macroplastics were exposed to constant wind speeds that simulated gentle to moderate breezes (2.3, 2.7, and 3.2 m/s, which correspond to Beaufort 3–4) and ‘heavy’ rain intensities (1.4, 2.0, and 3.3 mm/min)^29^. Using object tracking software the displacement of the macroplastics was determined. Through experimental repetitions, macroplastic mobilization probabilities, defined as the fraction (%) of plastic items that had a detectable non-zero displacement, were computed. For all mobilized plastic items, the transport velocity was calculated by dividing the total displacement by the total duration of the movement.

Here, we present our empirically derived wind- and rain-driven mobilization probabilities and transport velocities of macroplastics on land. We demonstrate their dependence on the terrain characteristics and type of plastic waste. We show the relationships that we observed between the applied wind speed and rain intensity and the resulting macroplastic transport velocity. We conclude that the movement of plastic waste on land, driven by natural forces, is a complex interplay between material and terrain characteristics. In our experimental setting, wind had a higher mobilizing and transport driving capacity than heavy rainfall. Therefore, we advocate that models should not relate macroplastic transport on land to only rain intensity, but rather include factors that determine the development of surface runoff with favorable flow dynamics. However, future research on surface runoff development needs to validate this outcome. Altogether, we anticipate that our empirical dataset on wind- and rain-driven macroplastic mobilization and transport on land will have a wide applicability in modelling and monitoring studies.

## Results

### Macroplastic mobilization probability

#### Macroplastic properties determine mobilization probability

**Figure 1:**
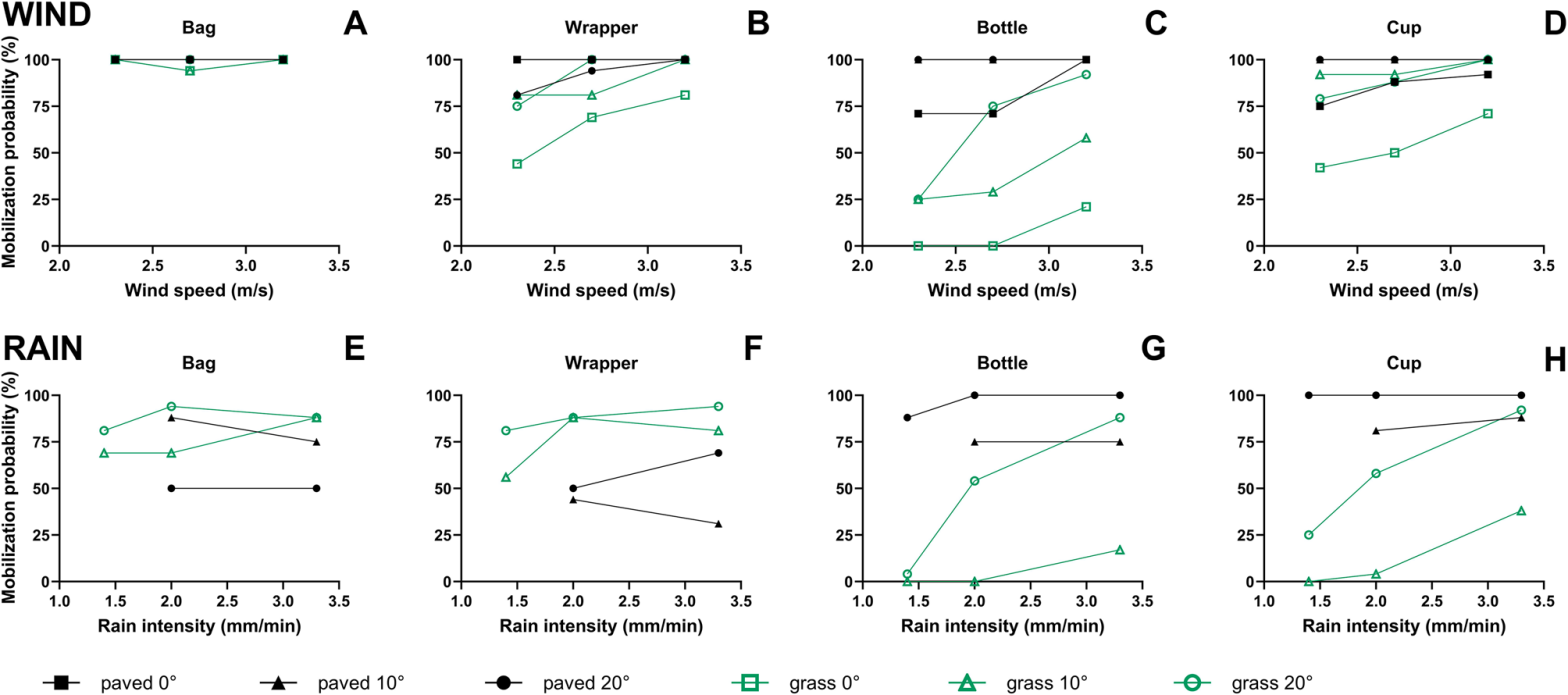
Wind- and rain-driven macroplastic mobilization probabilities (%) of bags, wrappers, bottles and cups on paved and grass terrains with 0°, 10° and 20° slopes. Applied wind speeds were 2.3, 2.7 and 3.2 m/s (measured 0.12 m above surface). Applied rain intensities were 1.4, 2.0 and 3.3 mm/min. Purely gravity-driven mobilization on slopes (i.e., for which no wind or rain force was required) is not included in this data. The total number of tested plastic items, i.e., 100%, corresponds to 16 (in panels **A**,**B**,**E**,**F**), 24 for ‘paved 0°’/‘grass 0°’/‘grass 10°’/‘grass 20°’ and 16 for ‘paved 10°’/‘paved 20°’ (in panels **C** and **D**), and 16 for ‘paved 10°’/‘paved 20°’ and 24 ‘grass 10°’/‘grass 20°’ (in panels **G** and **H**). The data plotted here is also provided in Tables S1 and S2.

The macroplastic items that we used in our experiments (bags, wrappers, bottles, and cups) have different material properties (e.g., size, shape, density, mass, and flexibility/rigidness). We observed distinct mobilization probabilities for the four types of macroplastic while keeping driving forces (e.g., wind speed or rain intensity) and terrain characteristics constant. For example, on a 0° grass surface a wind speed of 2.3 m/s mobilized 100% of the bags, 44% of the wrappers, 42% of the cups, and none of the bottles (Fig. 1A,B,C,D). Ranking the four types of macroplastics from highest to lowest average mobilization probability resulted, for both wind and rain, in: bags, wrappers, cups and bottles.

#### Terrain slope plays larger role in rain-than in wind-driven mobilization

We tested 0°, 10° and 20° terrain slope angles to examine whether the terrain slope affects the mobilization of macroplastics on land. We found that rain-driven macroplastic mobilization probabilities increased with terrain slope angle (rain experiments were only performed on 10° and 20°). The rain-driven mobilization probability of plastics on a paved surface exposed to a 3.3 mm/min rain intensity, increased with a factor 2.2, 1.3, and 1.1, upon a slope angle change from 10° to 20° for wrappers, bottles, and cups, respectively. On grass these factors were: 1.2, 5.3, and 2.4, respectively (Fig. 1F,G,H). For bags, the slope increment from 10° to 20° resulted in a lower (factor 0.7) mobilization probability on the paved surface and in the same mobilization probability on the grass (for the 3.3 mm/min rain intensity; Fig. 1E). Driven by wind, a positive relationship between mobilization probability and terrain slope was only observed for bottles and cups (Fig. 1C,D). Overall, our results indicated that terrain slope plays a larger role in rain- than in wind-driven mobilization of macroplastics on land.

#### Mobilization increases for lower surface roughness

To examine the effect of terrain roughness on the mobilization of macroplastics on land, we used a smooth paved surface (very low surface roughness) and grass (medium surface roughness). Our experiments demonstrated that, in general, the macroplastic mobilization probability was higher on the paved surface than on grass (Fig. 1). Exceptions were bags mobilized by wind (Fig. 1A), which appeared independent of the terrain roughness, and bags and wrappers mobilized by rain, which had highest mobilization probabilities on grass (Fig. 1E,F). Driven by wind, the mobilization probability was on average 1.5 times higher on the paved surface than on grass. The mobilization probability of bottles and cups driven by rain was on average 6.3 times higher on the paved surface than on grass. In summary, the mobilization probability of macroplastics is in general higher on terrains with a lower surface roughness.

#### Mobilization probability increases with magnitude of driving force

To investigate the relationship between macroplastic mobilization probability and wind speed or rain intensity, three wind speeds and rain intensities were tested. We found that, in general, both higher wind speeds and higher rain intensities increased the fraction of mobilized macroplastic items (Fig. 1). After each rain experiment, we observed small accumulations of water in patches on the bags and wrappers. On the bottles and cups individual droplets of water were observed on their surfaces. For all three tested wind speeds, the mobilization probability of bags was 100%, except on grass where for the 2.7 m/s wind speed, mobilization was 94% (Fig. 1A). The rain intensity increment from 2.0 to 3.3 mm/min sometimes caused a counterintuitive decrease in the mobilization probability of bags and wrappers (Fig. 1E,F). In the Discussion section we examine the potential underlying factors for this. Overall, macroplastic mobilization probabilities increased with wind speed and rain intensity.

### Macroplastic transport velocity

Macroplastic transport velocities driven by wind and rain were computed for the mobilized items only. Here we present our findings regarding the relationships between macroplastic transport velocity and four variables:

(i) the type of macroplastic
(ii) the terrain surface roughness
(iii) the terrain slope
(iv) the driving force magnitude

When focusing on the relationship between macroplastic transport velocity and one of these variables, the transport velocities were pooled for the remaining three variables. We refer to Tables S1 and S2 for the macroplastic transport velocities that were found for all individual combinations of the four variables.

#### Transport velocity varies between different types of macroplastic

**Figure 2:**
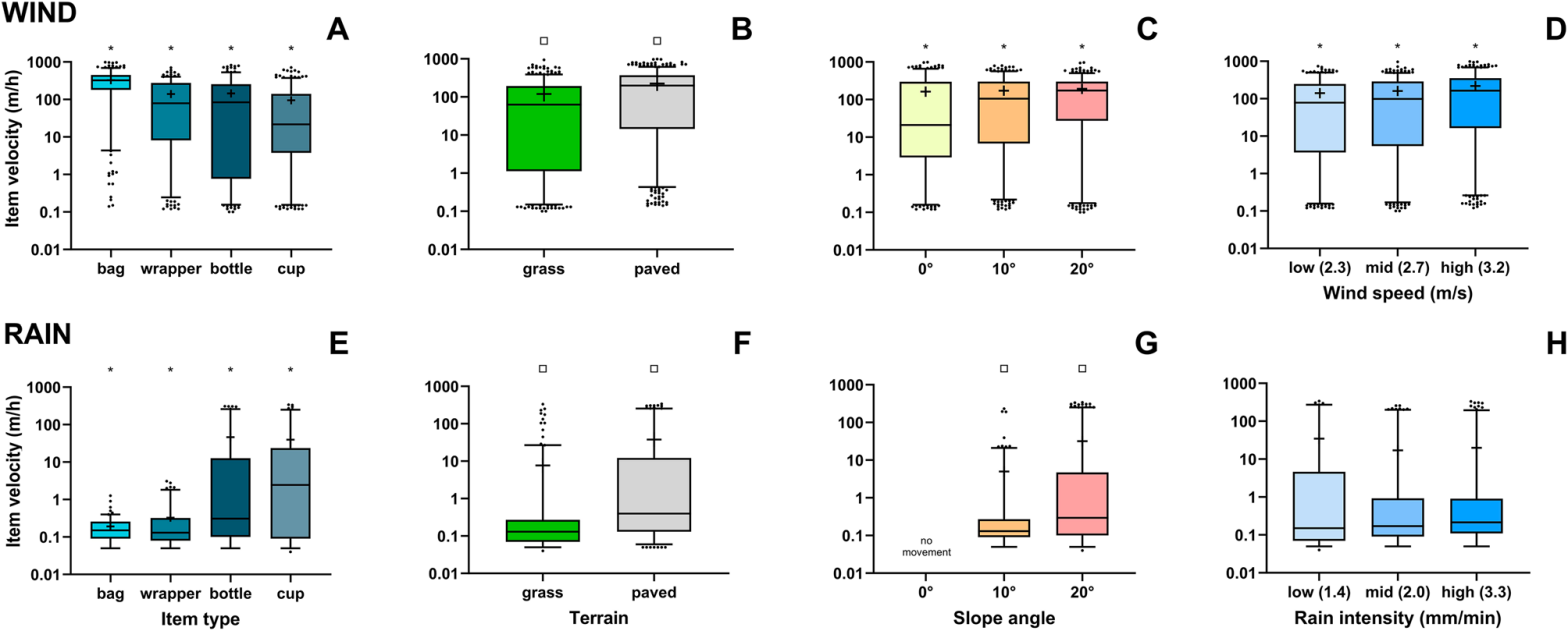
Transport velocities (m/h) of the macroplastics that were mobilized by wind (**A**–**D**) and rain (**E**–**H**). Boxes and whiskers show the 25–75% and 5–95% percentiles, respectively. Mean and median transport velocities are indicated with a ‘ + ’ and a horizontal line, respectively. Significantly different medians (Kruskal–Wallis test for three or more groups) or means (Mann–Whitney test for two groups) between groups are indicated by an asterisk (*) or a square (□), respectively.

The four different types of macroplastics displayed significantly different medians of transport velocities (p < 0.0001). We found the following mean standard deviation wind-driven transport velocities: 328 ± 205 m/h (bags), 145 ± 184 m/h (wrappers), 140 ± 152 m/h (bottles), and 94 ± 134 m/h (cups; Fig. 2A). For rain-driven transport we found the following mean velocities: 46 ± 91 m/h (bottles), 39 ± 82 m/h (cups), 0.33 ± 0.54 m/h (wrappers), and 0.19 ± 0.16 m/h (bags; Fig. 2E). Our results demonstrated that wind- and rain-driven transport velocities of the same type of macroplastic can differ up to two orders of magnitude. Bags, for example, had the highest wind-driven mean transport velocity, but the lowest rain-driven mean transport velocity. In summary, the transport velocity of macroplastics depends on the type of plastic and whether it is driven by wind or rain.

#### Transport velocity higher on smooth terrain

The mean macroplastic transport velocities, driven by wind or rain, were significantly higher on the paved surface than on the grass (p < 0.0001; Fig. 2B,F). The mean wind-driven transport velocity on the paved surface (225 ± 213 m/h) was 1.9 times higher than the mean wind-driven transport velocity on the grass (119 ± 148 m/h; Fig. 2B). For the rain-driven transport, the mean transport velocity on the paved surface (38 ± 84 m/h) was 4.8 times higher than on the grass (8 ± 37 m/h; Fig. 2F). Our results demonstrated that macroplastic transport velocities on land are higher on terrains with a low(er) surface roughness.

#### Transport velocity positively correlated to terrain slope

To test the influence of the terrain slope on the transport velocity of macroplastics on land, we performed wind experiments on 0°, 10° and 20°, and rain experiments on 10° and 20°. Both wind- and rain-driven macroplastic median transport velocities significantly increased with the terrain slope (p < 0.0001; Fig. 2C,G). The increment in transport velocity per 10° slope angle was larger for rain- than for wind-driven transport. For rain-driven transport the mean transport velocity increased from 5 ± 27 m/h (10°) to 32 ± 78 m/h (20°), i.e,. a factor of 6.4. Wind-driven macroplastic transport mean values increased from 162 ± 220 m/h (0°), to 172 ± 187 m/h (10°), to 193 ± 171 m/h (20°), i.e., for both 10° slope angle increments a factor of 1.1.

#### Transport velocity significantly changes with increased wind speed, but not with rain intensity

We only found a significant difference of the median macroplastic transport velocities between the three applied wind speeds (p < 0.0001; Fig. 2D). The mean wind-driven transport velocities that we found for the tested wind speeds were: 141 ± 164 m/h (for 2.3 m/s), 161 ± 175 m/h (for 2.7 m/s), and 219 ± 221 m/h (for 3.2 m/s; Fig. 2D). Our rain experiments did not provide evidence of significant median macroplastics transport velocity differences between the applied rain intensities (p = 0.2177, Fig. 2H). We obtained the following mean rain-driven transport velocities for the tested rain intensities: 35 ± 86 m/h (for 1.4 mm/min), 17 ± 53 m/h (for 2.0 mm/min), and 20 ± 63 m/h (for 3.3 mm/min; Fig. 2H).

### Relationship between wind or rain force and macroplastic transport velocity

**Figure 3:**
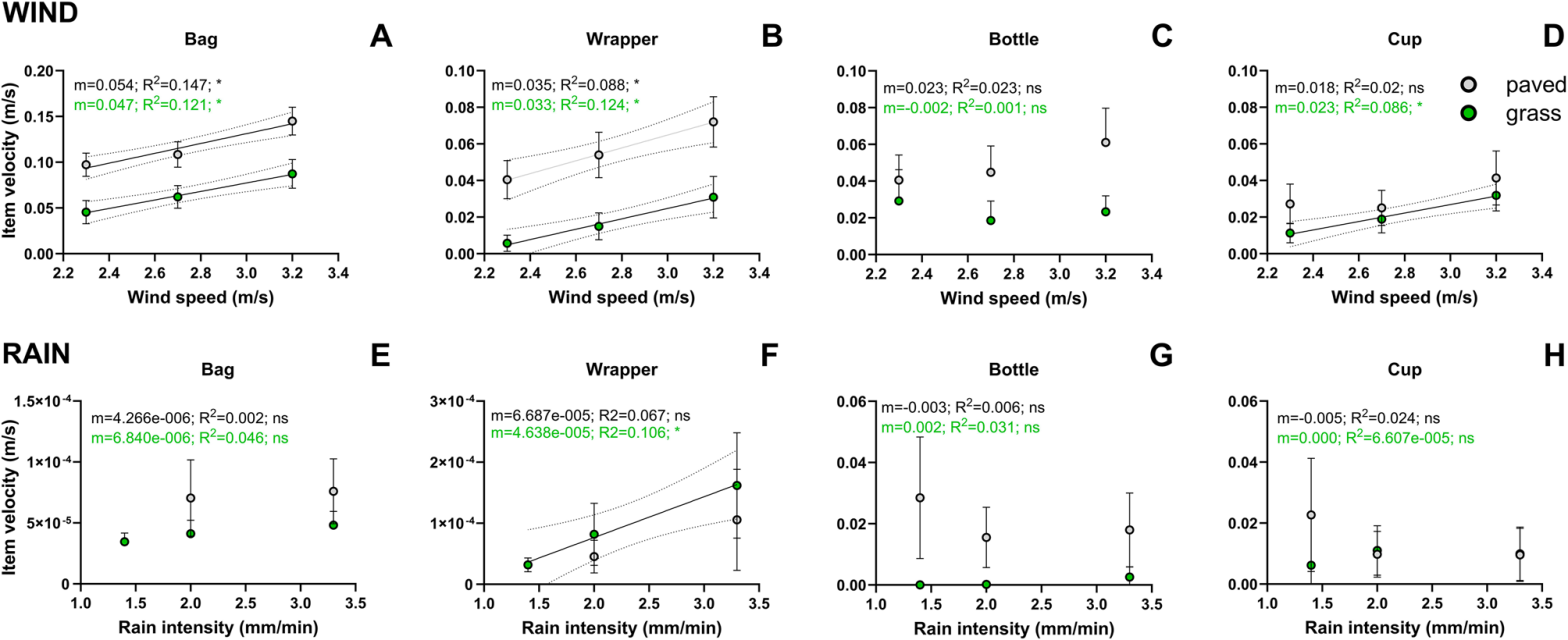
Average transport velocity (m/s) of the macroplastics that were mobilized in response to the applied wind speeds (**A**–**D**) and rain intensities (**E**–**H**). Linear regression models were fitted through all macroplastic transport velocities found per applied wind speed or rain intensity for each type of plastic item and for both the paved and grass surface. For this the macroplastic transport velocities on all terrain slope angles were combined. Solid lines show the significantly non-zero (*) fitted linear regression models. The slope (m) and the R^2^ of the linear models are provided at the top of each panel. The dotted lines indicate the 95% confidence interval of the linear model.

Combining all slopes, we observed significant positive relationships between macroplastic transport velocity and applied wind speed within the tested wind speed range (Fig. 3). The macroplastic transport velocity in response to the applied wind speed was highest for bags. On paved and grass surfaces, the bags adopted 5.4% and 4.7% of the applied wind speed, respectively (Fig. 3A). The transport velocity of wrappers was 3.5% of the applied wind speed on paved, and 3.3% on grass surfaces (Fig. 3B). The transport velocity of bottles was not significantly correlated to the applied wind speed (Fig. 3C). For cups, only their transport velocity on grass was significantly correlated to the wind speed (Fig. 3D). Rain-driven macroplastic transport velocities were, except for wrappers on grass (Fig. 3F), not significantly correlated to the applied rain intensity (Fig. 3E,G,H).

### Comparison with model studies

**Figure 4:**
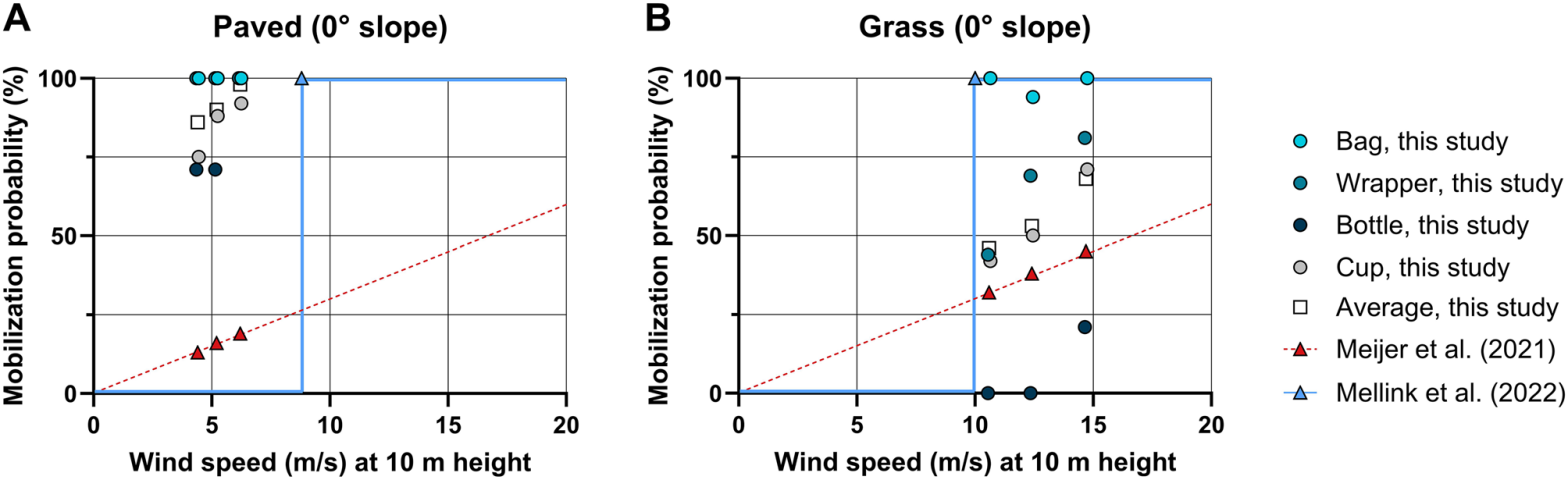
Wind-driven macroplastic mobilization probabilities (%) of bags, wrappers, bottles and cups on paved surfaces (**A**) and grass (**B**) with a 0° terrain slope angle. Average mobilization probabilities of all four plastic items are indicated by an ‘□’. Wind speeds (m/s) were translated from the experimental setup of 0.12 m above ground to the standard height of 10 m above the ground using the log wind profile equation (for details see “Comparison with other model studies”). Due to the difference in surface roughness, this corresponds to wind speeds of 4.4, 5.2 and 6.2 m/s for the paved surfaces (**A**) and 10.6, 12.4, and 14.7 m/s for the grass (**B**). The dotted red and solid green curve show the wind-driven mobilization probability as simulated in the model by Meijer et al.^27^ and Mellink et al.^28^, respectively.

We compared the wind-driven mobilization probabilities that we found in our experiments on the 0° paved and grass surfaces with the ones used in the models by Meijer et al.^27^ and Mellink et al.^28^. This comparison showed that, except for bottles on grass, our wind-driven mobilization probabilities were higher than modelled by Meijer et al.^27^ (Fig. 4A,B). Our average mobilization probabilities of all four items were at least 5 (for paved; Fig. 4A) and 1.4 (for grass; Fig. 4B) times higher than modelled by Meijer et al.^27^. The 8.8 m/s and 10 m/s wind thresholds used in the model from Mellink et al.^28^ mark the wind speeds above which 100% mobilization occurs on paved surfaces (Fig. 4A) and grass (Fig. 4B), respectively. Their model assumed 0% macroplastic mobilization for wind speeds below those thresholds. Our experiments showed that all four types of macroplastic already had a mobilization probability > 70% on a paved surface when exposed to wind speeds below 8.8 m/s (Fig. 4A). Our mobilization probabilities on grass were, except for bags, lower than 100% for wind speeds above the wind speed threshold of 10 m/s assumed by Mellink et al.^28^. In fact, bottles required a wind speed higher than 12.4 m/s to mobilize at all (Fig. 4B).

## Discussion

The mobilization and transport of macroplastics can be considered as the net result of a force balance between driving and resisting forces (the relevant forces are described in SI1). Our wind experiment results comply with physics principles: stronger winds increase macroplastic mobilization and transport; macroplastics with relatively large surface areas compared to their mass move faster; low-friction terrains and steep slopes enhance macroplastic mobilization and transport. While increasing wind velocity correlates positively with macroplastic mobilization and transport, increasing rain intensity does not exhibit a similar effect. This implies that rain intensity (or cumulative amount of rain) is not the primary driver of macroplastic mobilization and transport. Instead, we believe that the controlling factor is surface runoff. In the absence of surface runoff, mobilization or transport of macroplastics on land is unlikely, regardless of the rain intensity. Although surface runoff developed to a small degree in our experiments, it did not have the hydraulic characteristics required to mobilize macroplastics. We speculate that the surface runoff must be a sufficiently deep water layer to enable positively buoyant plastics to become and remain afloat. In our rain experiments, such favorable surface runoff conditions did not develop, presumably due to the steepness of the tested slopes (10° and 20°) and the absence of topographical depressions or gullies. Rather than being uplifted and transported afloat, the rain only made the plastic items wet. As a result, the gravitational force exerted by the accumulated water mass pressed the plastic items downward onto the surface. This impacted the mobility of especially the flexible macroplastics (e.g., bags and wrappers), as they accumulated more water in confined puddles on their surface compared to the more rigid plastics (e.g., bottles and cups). Our data counterintuitively suggests that bags on a paved surface with a 20° slope have a lower mobilization probability than bags on a 10° slope. Further insights will be required to explain this outcome. Our experiments demonstrate that the mobilization and transport of macroplastics, influenced by various forces, involve complex dynamics; while wind-driven processes align with expected force relationships, rain-driven mobilization and transport seems mainly governed by the flow dynamics of the resulting surface runoff rather than the rain intensity.

The dataset presented in this study holds important implications for the development of macroplastic transport and land-to-water emission models. Our experiments highlight the critical influence of terrain characteristics and material properties on macroplastic mobilization and transport, necessitating their incorporation into models. A comparative analysis with two recent models (Meijer et al.^27^ and Mellink et al.^28^) reveals that linear relationships or thresholds alone are insufficient to fully capture the dynamic nature of macroplastic mobilization on land. Our results suggest that models should not relate macroplastic transport on land to only rain intensity or cumulative rainfall, but rather include factors that determine the development of surface runoff with favorable flow dynamics, such as a deep water layer and high flow velocities. Using the wind-driven mobilization probabilities and transport velocities derived from our study, models can calculate macroplastic travel distances based on spatiotemporal wind data. A notable advantage of this approach is the elimination of reliance on model grid resolution, a limitation present in the models from Meijer et al.^27^ and Mellink et al.^28^ that compute macroplastic movement between adjacent model grid cells. The implementation of terrain and material property dependent macroplastic mobilization and transport dynamics will improve modeled predictions on macroplastic transport and accumulation on land, and emissions from land-based sources into rivers and oceans.

The pronounced impact of macroplastic material properties on their mobilization and transport on land can offer insights into the prevalence of specific items in natural environments. For instance, our experiments reveal particularly high wind-driven mobilization and transport velocities for dry plastic bags. This indicates that dry plastic bags, and possibly other flexible polyolefins (commonly assigned to a category called “PO_soft_”) have a substantial potential to be transported over large distances from their original land-based sources. This could explain why PO_soft_ items are frequently reported as the dominant plastic category afloat in rivers^30,31^ and accumulated on riverbanks^32–34^. Studies by Heo et al.^11^ and Imhof et al.^13^ have similarly suggested that spatial variabilities in plastic type distributions could be caused by selective wind-driven transport. We illustrate the concept of selective macroplastic mobilization and transport based on the type of plastic in Fig. S2. Recognizing that the distance mismanaged macroplastic waste travels from its land-based source depends on the type of plastic, can help to elucidate macroplastic abundances found in the natural environment and inform policymakers to prioritize interventions targeting highly mobile types of macroplastics.

The experimental design of this study had several limitations. Firstly, macroplastics were not exposed to wind and rain simultaneously, a condition commonly occurring in the natural environment. We anticipate that the mobilization probability and transport velocity of wet macroplastics are lower than for dry ones. The additional mass of water was observed to hinder rain-driven mobilization and transport, and is therefore expected to also hinder wind-driven mobilization and transport. Consequently, the wind-driven mobilization probabilities and transport velocities reported in this studye likely only apply to dry items and may overestimate those for wet items. Secondly, the absence of obstacles on the here used artificial surface does not reflect real-world scenarios where vegetation can entrap macroplastics^35^ or buildings can obstruct macroplastics in a way similar to what has been observed for the transport of dust^36^ and sand^37^. Therefore, our reported transport velocities are applicable to open, obstacle-free terrains. Thirdly, the applied wind field was not consistent across the entire artificial surface; wind speeds decreased gradually with distance from the wind source. As a result, macroplastics moving away from the wind source experienced reduced wind speeds, potentially hindering their continued movement. Thus, the wind-driven macroplastic transport velocities we found might be slightly underestimated. Lastly, our experiments utilized pristine macroplastics, whereas macroplastics in natural environments can weather and become (partially) covered with sediments^3^. The added mass of sediments is likely to hinder wind- and rain-driven mobilization and transport. tThe precise impact of weathering processes and sediment remnants on the wind and rain conditions necessary for macroplastic mobilization and transport on land remains unclear.

We recommend the following for future research. Firstly, the macroplastic transport velocity relationships with wind speed and rain intensity that we present in this study only apply within the tested wind speed and rain intensity ranges. Whether these relationships continue beyond the tested ranges needs exploring. Secondly, the dimensions of the artificial surface that we used were rather small and we recommend to upscale these dimensions whereby field, rather than laboratory, experiments could also be considered. Thirdly, obstacle (e.g., vegetation or buildings) density potentially determines the distance that macroplastics can travel on land, and deserves to be explored in more detail. Additionally, our experiments could be repeated with weathered macroplastics, representative of field-collected plastics. This could improve our understanding of the impact of weathering processes on macroplastic material properties and subsequent mobilization and transport behavior on land. We recommend future experiments to encompass also soft, hard, and expanded polystyrene fragments of varying sizes to better represent another plastic type that is abundantly found in field studies (e.g., van Emmerik et al.^32^ and de Lange et al.^34^). Lastly, although our findings suggest a dominant role for wind-driven macroplastic displacement on land, further experimental investigations are necessary to comprehensively elucidate the hydraulic processes underlying rain-driven mobilization and transport of macroplastics in terrestrial environments. After all, ponding, surface runoff and extreme flood events are known key drivers of macroplastic transport on land^38,39^.

## Conclusions

In this study wind- and rain experiments were performed on an artificial hillslope to quantify the mobilization and transport of macroplastics on land. We find that macroplastic mobilization (1) is positively correlated to wind speed and rain intensity, and (2) depends on terrain characteristics and the type of plastic. Macroplastic transport velocities on land are (1) positively correlated to wind speed, (2) not correlated to rain intensity, (3) increase with decreasing surface roughness, and (4) increase with increasing terrain slope. Our experiments demonstrate that macroplastic type (bottle, wrapper, cup, or bag) play a key role in determining their wind- and rain-driven mobilization and transport velocity on land. The absence of a correlation between rain intensity and macroplastic transport, makes us believe that regardless of the amount of rain, macroplastic transport on land requires the development of surface runoff with flow dynamics that allow for bringing macroplastics afloat. Based on this, we recommend to re-think the correlations between amount of rain and macroplastic mobilization on land adopted in models. Our experiments have contributed to our fundamental understanding of the mobilization and transport dynamics of macroplastics on land. The dataset that we present can be implemented in (existing and future) models and improve estimates on macroplastic pathways on land and emissions to rivers. Empirically based macroplastic transport models are invaluable for policymakers to develop removal and prevention strategies that aim to reduce the threat of plastic pollution to ecosystem and human health.

## Methods

### Experimental set up

#### Artificial hillslope

**Figure 5:**
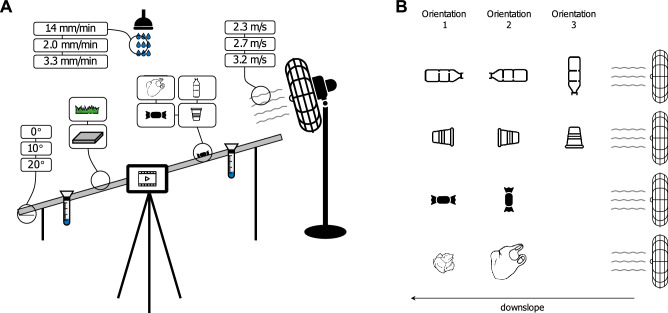
(**A**) Schematic side view of the experimental set-up of the artificial hillslope and the values for the variables that were tested. The rain experiments were only performed on 10° and 20° slopes. (**B**) Schematic top view of the relative macroplastic orientations with respect to the wind and downslope direction.

The experiments took place between June 2021 and January 2022 at the Kraijenhoff van de Leur Laboratory for Water and Sediment Dynamics at Wageningen University & Research, the Netherlands. An artificial hillslope of 60 by 120 cm was built of which the cover and slope angle were adjustable (Fig. 5A). Two terrain covers were created: paved and grass. The former consisted of eight 30 by 30 cm concrete tiles; the latter was a full coverage artificial grass mat with a maximum grass blade height of 3.5 cm. The tested terrain slope angles were 0°, 10°, and 20°.

#### Wind experiments

The wind flow was generated by an electrical wind stand fan (Fig. 5A). The fan had three rotating blades inside a metal mesh cap which had a diameter of 40 cm. An anemometer measured the wind speed in meters per second. For each terrain slope angle, the inclination of the wind fan cap was adjusted to ensure a wind flow direction parallel to the surface. After initiating the wind flow, it took on average 5 s to reach a constant wind speed. The wind fan generated three wind intensities. The wind fan was positioned in such a way that the plastic items were subjected to wind speeds of 2.3, 2.7, and 3.2 m/s. These wind speeds were measured at a height of 12 cm above the surface and 30 cm away from the longest and 15 cm away from the shortest side of the surface (Fig. S3). An evaluation of the spatial variations of the wind speed across the surface revealed that 90 cm behind the position of the anemometer the wind speed had decreased to ~ 1 m/s.

#### Rain experiments

On flat terrains the gravity component parallel to the surface equals zero. Therefore, in theory, water on a flat terrain will not flow. Hence it was assumed that (flowing) surface runoff will not develop on flat terrains. For that reason, rain experiments were only performed on terrain slope angles of 10° and 20°. The rain was generated by a rainfall simulator^40^ featuring two nozzles at 5 m above the surface (Fig. 5A). Four rain gauges were installed at the corners of the surface to measure the amount of rainfall (mm). The mean amount of rain in the four gauges after 5 min was used to calculate the rainfall intensity (mm/min). Three rain intensities were generated: 1.4, 2.0, and 3.3 mm/min. These correspond to intensities of 84, 120, and 198 mm per hour, which fall under the category ‘heavy’ rainfall events^29^. A part of the surface runoff flowed below the concrete tiles and the grass mat, because of the open fissures between the concrete tiles and the permeable grass mat. Both the upper- and subsurface runoff was collected at the lower end of the surface.

#### Macroplastics

Four plastic item types (20 items for each plastic type) were used in the experiments: 0.5 L water bottles without cap (mass: 13.6 g, polymer: PET), 0.25 L drinking cups (mass: 6.2 g, polymer: PET), wrappers of a chocolate candy bar (mass: 0.5 g, multilayer of PP and PE), and 25 L trash bags (mass: 2.1 g, polymer: LDPE) (Fig. S1). These plastic items were selected to assure a variety in shapes, densities, masses and flexibility/rigidness. Moreover, this item selection accounts for about half of the types of plastic litter found in and around rivers^30,32^.

#### Video recording

A GoPro HERO5 camera was used to record each experiment. The videos had a pixel resolution of 1080 by 1920 and a time resolution of 60 frames per second. The GoPro was installed on a tripod and positioned next to the artificial hillslope (Fig. 5A). The GoPro had an inclination of 30° with the horizon. The height of the GoPro and its distance to the surface was adjusted for each set up to ensure that the interesting part of the surface was captured in the video frame.

### Experiment protocol

At the start of each experiment the terrain cover and slope angle were established. Next, the camera was started to record immediately followed by placing two dry plastic items of the same type on the surface. The centers of the two plastic items were always placed at the same locations on the surface: 15 cm away from the longest side and 45 cm away from the shortest side of the surface (Fig. S3). For each experiment the two plastic items had the same orientation relative to the wind flow and downslope direction. For bottles and cups, three different item orientations were tested: with the opening in the opposite direction of (orientation 1), in the same direction as (orientation 2), and perpendicular to the direction of (orientation 3) the wind flow and downslope (Fig. 5B). For the wrappers, experiments were performed for two different item orientations: with the longest axes of the wrapper parallel (orientation 1) and perpendicular to (orientation 2) the direction of the wind flow and downslope (Fig. 5B). The bag ‘orientations’ were crumpled up (orientation 1) and unfolded (orientation 2) (Fig. 5B). The reason for testing different item orientations was to investigate whether the shape of the plastic item in combination with the direction of the applied wind flow and terrain slope, had an impact on the mobilization probability and transport velocity of the plastic item. If the plastic items, after placing them on the surface, immediately moved (i.e., rolled) downslope due to gravity, then no wind or rain force was applied. If the plastic items steadily rested on the surface, then first the highest wind speed or rain intensity was applied. The wind or rain force was applied until both plastic items fell off the surface, or after 2 min for the wind, and 5 min for the rain experiments. At the end of the experiment the video recording was stopped. After each rain experiment the paved surface was briefly wiped with a towel to remove any stagnant water. Each experiment was repeated four times (one experiment tested two items simultaneously, so the total number of tested items per experiment was eight). At the end of each experiment a ruler was used to check whether the plastic items had moved away from their initial start positions. In case all eight plastic items were not displaced by a certain wind speed or rain intensity, then it was assumed that a lower wind speed or /rain intensity would neither displace the items. Therefore, in those cases, follow-up experiments with a lower wind speed or rain intensity were not performed and a zero displacement was assumed.

### Data analysis

The videos of all experiments were analyzed with the open source software Kinovea^41^. This software uses color and contrast differences to track the position of an object throughout a video. In the software environment, a marker was placed on a detectable part of the plastic item. For the bottle this was on the blue ring at its neck, for the wrapper on a printed logo, for the cup on a drawn circle, and for the bag on one of the drawn black dots. Next, the software tracked the x and y pixel coordinates of this marker through time. We refer to SI2 for further details on the analysis procedure that we performed in Kinovea The GoPro camera recorded the experiments from an inclined angle, which led to a distorted image. For this reason, the pixel coordinates had to be converted to real-world coordinates. For this conversion the Matlab function *fitgeotrans* was used whereby four adhesive putties at the edges of the artificial surface served as reference points. For further details on the coordinate conversion we refer to SI3.

When analyzing the data, all tested plastic item orientations were combined for each macroplastic type (see SI4 for extended results on the differences observed for the different macroplastic orientations). Purely gravity-driven mobilization of macroplastics (e.g., a bottle rolling down a terrain slope of 10°) was excluded from further analyses as it was out of scope of this study. The total displacement of a plastic item was defined by the difference between its start and end location on the surface. Bags exposed to rain tended to flatten-out due to the mass of the rainwater. Therefore, the location of a bag was defined by the coordinates of its midpoint, which lied halfway the outer most left and right stretch of the bag. The displacement vector was split into a component parallel ($x$) and perpendicular ($y$) to the wind and downslope direction (Fig. S3). With the aim to investigate the displacement of plastics triggered by an applied wind or rain force, this study only considered the component of the displacement that was parallel ($x$) to the wind and rain force.

Accuracy tests within the Kinovea software revealed that manually placing a marker repeatedly (n = 32) on the exact same spot on a plastic item was associated with an mean error of about 2 mm in real-world coordinates. Taking this uncertainty into account, plastic items that had an $x$ displacement of at least 4 mm (two times the uncertainty) were regarded as ‘mobilized’. All plastic items with an $x$ displacement of less than 4 mm were interpreted as ‘not mobilized’. The mobilization probability of a plastic item type on a specific terrain was defined as the percentage of plastic items that were mobilized under a given wind or rain force. As each wind and rain force was applied to eight plastic items in total, a mobilization probability of 100% meant that all eight items were mobilized at least 4 mm within 2 (wind) or 5 (rain) minutes. To focus on the mobilization capacity of the wind and rain force, mobilizations purely due to the force of gravity were left out of the mobilization probability computations. For all mobilized plastic items, the plastic item transport velocity was calculated by dividing the total $x$ displacement (in real-world coordinates) by the total duration of the movement. The unit of meters per hour was used to express the plastic item transport velocities, since river basin scale models commonly work with temporal and spatial resolutions in that order of magnitude.

After performing the Shapiro–Wilk, D'Agostino & Pearson, Anderson–Darling and Kolmogorov–Smirnov tests it was established that the plastic transport velocity distributions, as well as the residuals, were not normally distributed. Therefore, the nonparametric Mann–Whitney test (two groups, means) and the Kruskal–Wallis test (three or four groups, medians) were used to explore whether plastic transport velocities were significantly different for different wind speeds, rain intensities, terrain covers, terrain slope angles or plastic item types. Statistics were performed using GraphPad Prism, Version 9.4.1 (681).

### Comparison with other model studies

We compared the wind-driven macroplastic mobilization probabilities that we found with the ones used in the model studies from Meijer et al.^27^ and Mellink et al.^28^. The Meijer model assumes a linear relationship between the macroplastic mobilization probability and wind speed according to: $P\left({M}_{W}\right)=\left(\frac{1}{32.7}\right)* u *100\%$, with $P({M}_{W})$ in % and $u$ in m/s. This relationship is used for all land uses and terrain slopes (Meijer et al.^27^ only takes terrain characteristics into account for the computation of the probability of transport from land to river). The Plastic Pathfinder model from Mellink et al.^28^ uses a threshold approach in which pre-defined wind thresholds, which depend on the terrain characteristics, need to be exceeded in order to mobilize 100% of the macroplastics (Table 1 in Mellink et al.^28^). Both the Meijer^27^ and Mellink^28^ model use data on wind speeds that are measured at 10 m height above the surface. Since our wind speeds were measured at 0.12 m above the surface, we extrapolated our wind speeds to 10 m height. For this we used the log wind profile equation under the assumption of neutral stability conditions^42^, with which the mean wind speed at one height, $u\left({z}_{2}\right)$ (m/s), can be estimated based on that at another height, $u\left({z}_{1}\right)$ (m/s):

$$u\left({z}_{2}\right)=u\left({z}_{1}\right) \frac{{\text{ln}}\left(\left({z}_{2}-d\right)/{z}_{0}\right)}{{\text{ln}}\left(\left({z}_{1}-d\right)/{z}_{0}\right)}$$

with $d$ the zero plane displacement height (m) and ${z}_{0}$ the surface roughness (m). The zero plane displacement height is the height in meters above the ground at which zero mean wind speed is achieved as a result of wind-slowing obstacles (e.g., trees or buildings). We assumed $d = 0$, because of the absence of wind-slowing obstacles in our experiments. For paved surfaces a surface roughness of 0.001 m was adopted. For grass we assumed that the surface roughness is equal to the maximum blade height of the grass matt we used, i.e., 0.035 m. Table 1 shows the computed wind speeds at 10 m height for the three applied wind speeds for the paved surface and the grass.

**Table 1:** Wind speeds (m/s) at 0.12 m above the surface ($u\left({z}_{1}\right)$) extrapolated to wind speeds (m/s) at 10 m above the surface ($u\left({z}_{2}\right)$). For this extrapolation the log wind profile equation was used.

$u\left({z}_{1}\right)$ (m/s)	$u\left({z}_{2}\right)$	
	Paved terrain (m/s)	Grass (m/s)
2.3	4.4	10.6
2.7	5.2	12.4
3.2	6.2	14.7

We compared the rain-driven macroplastic mobilization probabilities that we found only with the ones used in the Plastic Pathfinder model from Mellink et al.^28^, because the units of the rain force were similar: mm/min and mm/day, respectively. In the Meijer model^27^ on the other hand, the annual cumulative amount of rain is used to estimate the rain-driven macroplastic mobilization probability: $P\left({M}_{R}\right)= 1.5*{10}^{-4} * RI*100\%$, with $P({M}_{R})$ in % and $RI$ in mm/year. We recognized that in order to make a meaningful comparison, we had to make too many assumptions to convert our tested rain intensities to annual cumulative amounts of rain.

### Supplementary Information

Supplementary Information.

## Data Availability

The datasets generated during and/or analyzed during the current study are available in the “Wind- and rain-driven macroplastic mobilization and transport on land” repository at 4TU.ResearchData, 10.4121/0be15175-28c1-44c5-a153-9d086308da6a.v1. All experiment videos are available upon request by contacting the corresponding author via email.
