# Supplementary material for: Wind- and rain-driven macroplastic mobilization and transport on land
Source: Sci Rep. 2024 Feb 16;14:3898. doi: 10.1038/s41598-024-53971-8 (PMC10873394; doi:10.1038/s41598-024-53971-8)
Supplement: Supplementary file 1 — Supplementary Information. [file 41598_2024_53971_MOESM1_ESM.pdf]

Wind- and rain-driven macroplastic mobilization and transport on land – *Supplementary Information*

Yvette A.M. Mellink[1], Tim H.M. van Emmerik[1] & Thomas Mani[2]

[1]Hydrology and Environmental Hydraulics Group, Wageningen University and Research, Wageningen, the Netherlands.
[2]The Ocean Cleanup, Rotterdam, the Netherlands.

Corresponding author: Yvette Mellink (yvette.mellink@wur.nl)

Table of contents

Supplementary Chapters

SI1:	Forces acting on an item on a slope	4
SI2:	Instructions item tracking procedure in Kinovea	5
SI3:	Conversion from pixel coordinates to true coordinates	8
SI4:	Transport behaviour of macroplastics in different orientations	9

Supplementary Tables

Table S1.	Results from the wind experiments	11
Table S2.	Results from the rain experiments	12

Supplementary Figures

Figure S1.	The four macroplastic items	15
Figure S2.	The concept of selective macroplastic mobilization and transport on land	15
Figure S3.	Schematic top view of the experimental set up	16

SI1: Forces acting on an item on a slope

Mobilization and transport of plastic items on land is the result of a force balance between driving and resisting forces. The driving forces considered in this study are wind, rain and (inevitably) gravity. The following set of equations from fundamental physics are relevant for our study:

the total wind force (N) on a plastic object: $$F_{wind} = \left(\frac{1}{2} \times \rho_{air} \times v^2\right) \times A \quad (1)$$

the total rain force (N) on a plastic object: $$F_{rain} = \left(\frac{1}{2} \times \rho_{water} \times u^2\right) \times A \quad (2)$$

the static friction force (N) on a plastic object: $$F_{stat\,fric} = \mu_{stat} \times \cos(\theta) \times m \times g \quad (3)$$

the kinetic friction force (N) on a plastic object: $$F_{kin\,fric} = \mu_{kin} \times \cos(\theta) \times m \times g \quad (4)$$

the component of gravity parallel to the surface: $$F_{g_{//}} = \sin(\theta) \times m \times g \quad (5)$$

where ρ_{air} and ρ_{water} are the density (kg/m^3) of air and water, respectively, v the wind speed (m/s), u the flow velocity of the surface runoff (m/s), A the surface area of the plastic object on which the force acts, μ_{stat} and μ_{kin} the coefficients of static and kinetic friction, respectively, θ the terrain slope angle (°), m the mass of the plastic object (kg) and g the gravitational acceleration (m/s^2).

In our experiments, the component of gravity parallel to the surface always acted in the same direction as the driving forces. Therefore the conditions of mobilization and transport of plastics can be expressed as:

mobilization of plastic by wind occurs when: $$F_{wind} + F_{g_{//}} > F_{stat\,fric} \quad (6)$$

mobilization of plastic by rain occurs when: $$F_{rain} + F_{g_{//}} > F_{stat\,fric} \quad (7)$$

transport of plastic by wind occurs when: $$F_{wind} + F_{g_{//}} > F_{kin\,fric} \quad (8)$$

transport of plastic by rain occurs when: $$F_{rain} + F_{g_{//}} > F_{kin\,fric} \quad (9)$$

SI2: Instructions item tracking procedure in Kinovea

We executed the following steps to track plastic items in Kinovea:

1. Go to **File** and open a video.

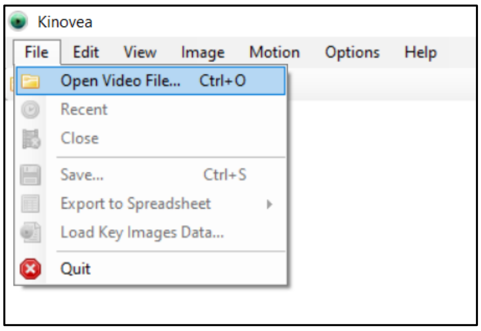

2. Click on the **Cross Marker '+'**.

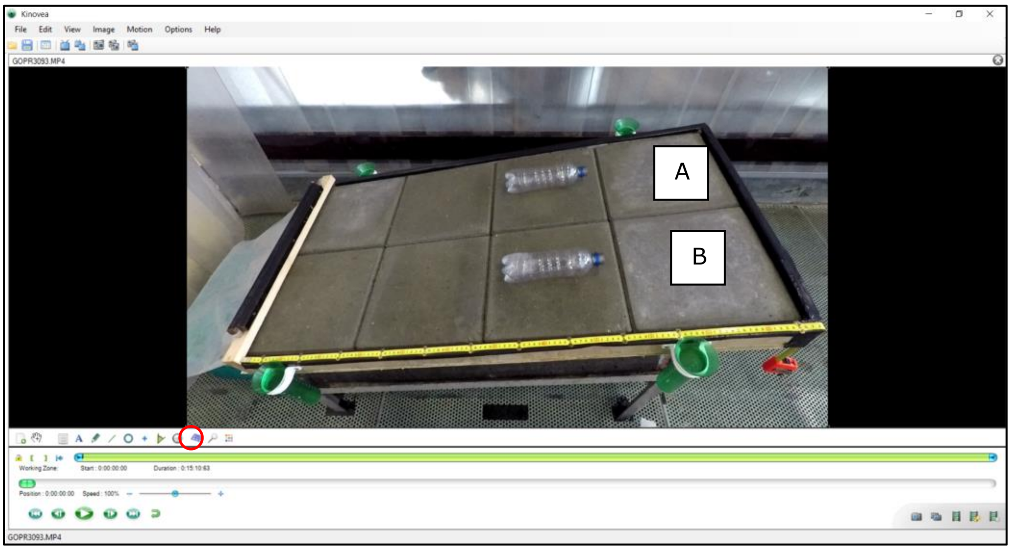

3. Place the cross marker (*left click*) somewhere on the plastic item. We used the following locations for the four plastic items:

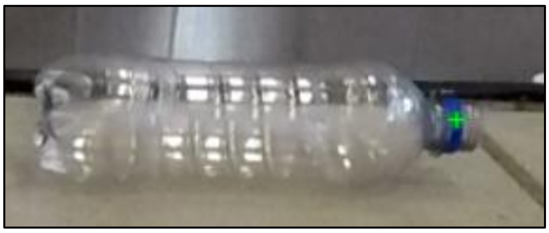

On the blue ring

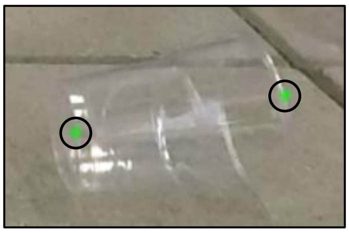

On the edges

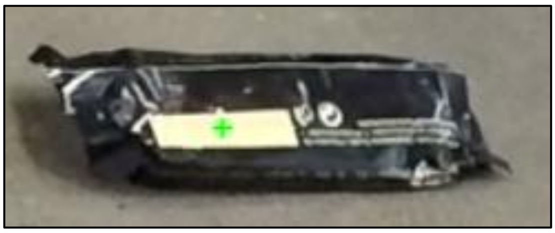

On the white square

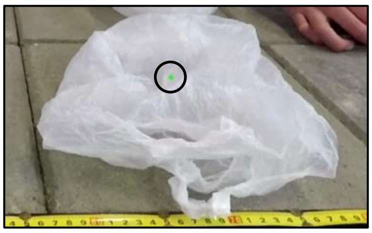

Somewhere in the middle

4. Right *click* on the cross marker and select **Display coordinates**. The first number shown in Kinovea is the pixel column coordinate and the second number is the pixel row coordinate.

5. Write down the initial pixel coordinates of plastic item. The pixel row number is a negative value, but write it down as a positive value (the Matlab code we developed will account for this).

6. Right click on the cross marker and select **Track Path**. Two **rectangular windows appear** around the cross marker.

7. Right click again on the cross marker and go to **Configuration**. The Configure Path window opens.

8. Select **Complete Path**

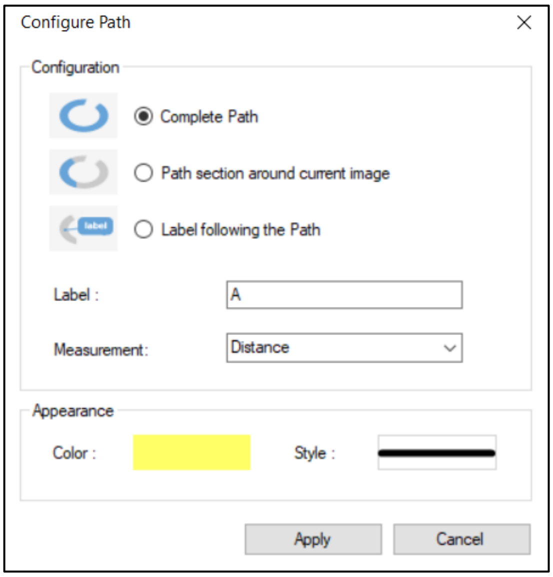

9. At **Label** write "A" in case this path belongs to plastic item A. Or "B" in case this path belongs to plastic item B.

10. At **Measurement** choose **Distance** from the drop down list.
11. Choose a colour that is clearly visible (e.g. yellow).

12. Click **Apply**.

13. Click on the green **Play** button 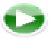 to start the tracking procedure.

14. Wait for Kinovea to track the position of the plastic item throughout the video.

15. Manually stop the tracking procedure when the plastic item falls off the side of the surface or hits the lower elevated edge on the left side of the slope

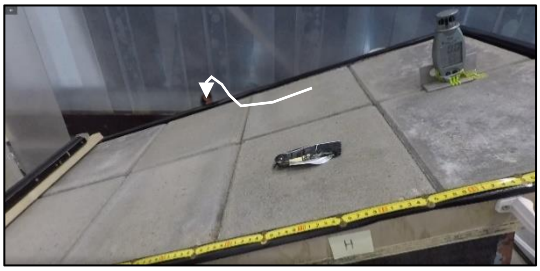

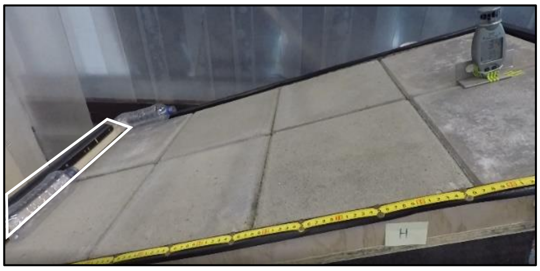

16. To stop the tracking procedure, click on the green **Pause button**

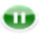

17. Finally, go to **File → Export to Spreadsheet → Microsoft Excel (MS-XML)**

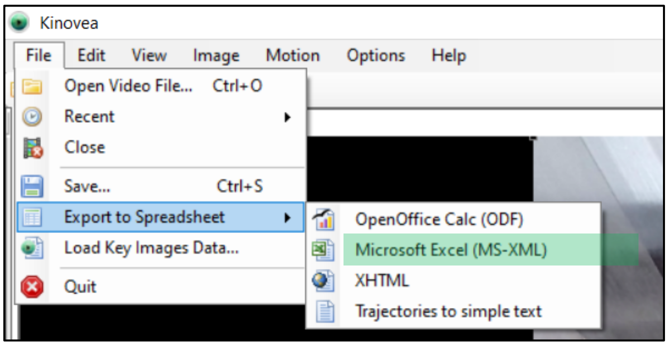

<u>SI3: Conversion from pixel coordinates to true coordinates</u>

In Matlab we wrote a short script (Coordinate_conversion.m; uploaded in the data repository of this publication), which can be used to convert the pixel coordinates of the path of the plastic items (stored in the output Excel file from Kinovea) to real-life coordinates (in mm). This conversion was necessary because the GoPro camera recorded the experiments from an inclined angle, which led to a distorted image (i.e. a square in real-life did not have a square shape in the video).

The pixel coordinate system that Kinovea applies to the video frames is depicted in Figure A(i). We multiplied the vertical pixel values with –1, to obtain positive pixel values. We refer to this 'new' coordinate system as the Matlab pixel coordinate system (Figure A(ii)). The Matlab script uses the function *fitgeotrans* to perform the conversion for each video. The *fitgeotrans* function uses two matrices called movingPoints and fixedPoints. The movingPoint matrix requires the pixel coordinates of four reference points in the video (provided in the Matlab pixel coordinate frame):

movingPoints = [UL_x UL_y; UR_x UR_y; LL_x LL_y; LR_x LR_y] (see Figure A(iii))

The values for the movingPoints matrices were determined manually in Kinovea for all camera positions and stored in an Excel file (which is called 'The bottles sheet.xlsx' in the example that we uploaded in the repository). The fixedPoints matrix contains the 'true' coordinates of the four reference points, in the same order as listed in the movingPoints matrix. In our case the fixedPoints matrix always contained the same values:

fixedPoints = [600 600 ; 0 600 ; 600 0 ; 0 0] (see Figure A(iv))

This is because our reference points were the four outer corners of four concrete tiles. For the grass experiments we placed putties on the edges of the surface that also were 600 mm apart.

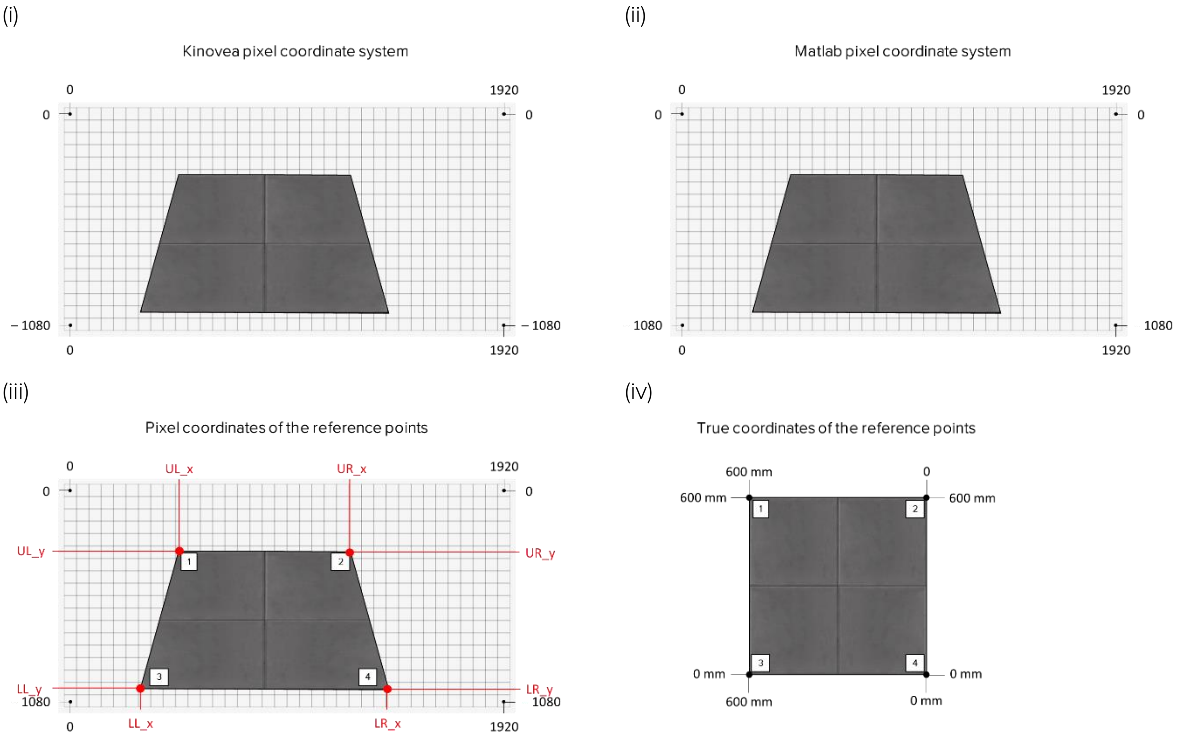

Figure A. Schematic drawings depicting the coordinate systems used in Kinovea (i) and in Matlab (ii). In the Matlab script called 'Coordinate_conversion.m' the pixel coordinates of four reference points in the video frame (iii) were written to the movingPoints matrix. The true coordinates of those four reference points, provided in mm, (iv) were written to the fixedPoints matrix.

SI4: Transport behaviour of macroplastics in different orientations

Bottles

For bottles, no considerable differences in transport dynamics were observed for orientations 1 and 2 in which the longest axis of the bottle was parallel to the wind flow and downslope direction. Bottles in orientation 3 on a 0° terrain had a higher mobilization probability and transport velocity compared to bottles in orientation 1 and 2. On sloping paved terrains, bottles in orientation 3 immediately rolled down under the force of gravity.

Drinking cups

For drinking cups on flat paved terrains, cups initially in orientation 1 and 3 re-oriented to orientation 2 as soon as the wind flow hit (Figure B). On sloping paved terrains the re-orientation process from 1 to 2 led to the cups rolling/stumbling down all the way to the lower end of the surface. Cups in orientation 3 on sloping paved terrains always immediately rolled/stumbles down purely due to the force of gravity (and were thus not included in the data anlysis). On grass terrains, re-orientation of cups was less frequently observed. Moreover, on sloping grass terrains cups in orientation 1 sometimes re-oriented to orientation 3 and continued to roll down all the way to the lower end of the surface in that orientation. On grass the force of gravity did not initiate cups in orientation 3 to roll down the slope immediately.

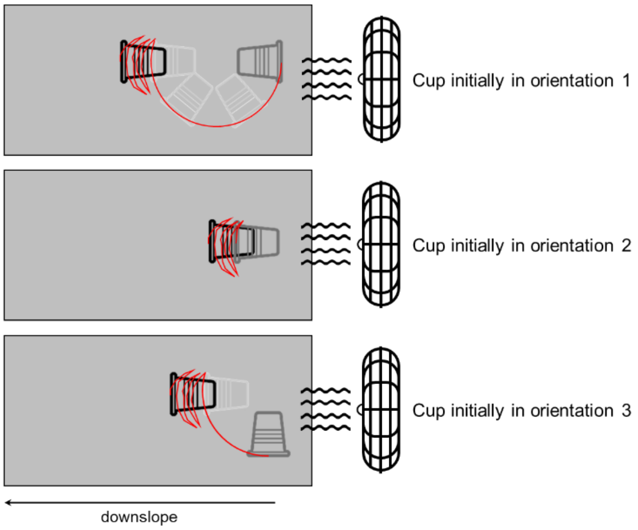

Figure B. Schematic top view depicting the behaviour of plastic drinking cups. It was observed that cups initially placed in orientation 1 and 3 re-oriented themselves to orientation 2 immediately after the wind or rain force was applied. Transport of cups in orientation 2 was characterized by halve circular wobbling along with the wind direction or downslope.

Food wrappers

For food wrappers, the mobilization probability and transport velocity was slightly higher when the longest axis of the wrapper was perpendicular to the wind flow and downslope direction (orientation 2). The mean transport velocity of food wrappers in orientation 2 was higher than for food wrappers in orientation 1 – twice as high in the case of rain-driven transport, and three times higher in the case of wind-driven transport.

Bags

For bags, orientation had a considerable impact not only on the mobilization probability and transport velocity, but as well on the mode of transport (note that in case of the bags orientation means their shape due to the state of crumpling). For example, the wind regularly lifted up the uncrumpled bags (orientation 2) from the terrain, thus reducing ground friction and making the transport velocity less depended on the type of terrain cover (paved vs grass). When exposed to rain on the other hand, the larger surface area of uncrumpled bags compared to crumpled-up (orientation 1) bags made them attain more rain water and thus gain more weight and ground friction. This lowered the mobilization probability and transport velocities of unfolded bags in the rain. The experiments confirmed the hypothesis that the shape of the plastic item in combination with the applied wind field and topography has an impact on its mobility and transport.

Supplementary Tables

Table S1. Results from the wind experiments. The mobilization probability, mean and median transport velocities (and standard deviation) are listed for all tested combinations of plastic item type, terrain type and slope angle. Transport velocities are expressed in meters per hour (m/h). Purely gravity driven transport of plastic items are excluded from the 'Total number of items tested'.

Macroplastic type	Terrain friction	Terrain slope angle (°)	Wind speed (m/s)	Total number of items tested	Total number of moved items	Mobilization probability (%)	Mean velocity of moved items (m/h)	Median velocity of moved items (m/h)	STD (m/h)
Bottle	Paved	0	2.3	24	17	70.8	215.1	0.6	232.9
Bottle	Paved	0	2.7	24	17	70.8	226.2	9.2	239.8
Bottle	Paved	0	3.2	24	24	100.0	272.9	25.1	336.5
Bottle	Paved	10	2.3	16	16	100.0	44.9	1.0	98.1
Bottle	Paved	10	2.7	16	16	100.0	26.8	2.5	83.6
Bottle	Paved	10	3.2	16	16	100.0	80.9	6.6	117.0
Bottle	Paved	20	2.3	16	16	100.0	173.2	174.1	70.6
Bottle	Paved	20	2.7	16	16	100.0	226.8	231.0	47.8
Bottle	Paved	20	3.2	16	16	100.0	279.5	295.3	73.1
Bottle	Grass	0	2.3	24	0	0.0	*n/a*	*n/a*	*n/a*
Bottle	Grass	0	2.7	24	0	0.0	*n/a*	*n/a*	*n/a*
Bottle	Grass	0	3.2	24	5	20.8	0.5	0.4	0.3
Bottle	Grass	10	2.3	24	6	25.0	58.2	46.7	53.2
Bottle	Grass	10	2.7	24	7	29.2	54.3	38.5	56.4
Bottle	Grass	10	3.2	24	14	58.3	107.1	123.9	72.8
Bottle	Grass	20	2.3	24	6	25.0	152.1	150.0	98.9
Bottle	Grass	20	2.7	24	18	75.0	71.5	0.9	100.6
Bottle	Grass	20	3.2	24	22	91.7	87.9	54.7	111.6
Food wrapper	Paved	0	2.3	16	16	100.0	148.0	138.8	133.5
Food wrapper	Paved	0	2.7	16	16	100.0	221.0	191.2	151.0
Food wrapper	Paved	0	3.2	16	16	100.0	302.1	287.6	177.2
Food wrapper	Paved	10	2.3	16	16	100.0	154.3	142.4	133.0
Food wrapper	Paved	10	2.7	16	16	100.0	171.3	199.4	126.8
Food wrapper	Paved	10	3.2	16	16	100.0	307.7	346.3	144.4
Food wrapper	Paved	20	2.3	16	13	81.3	132.2	142.5	94.4
Food wrapper	Paved	20	2.7	16	15	93.8	189.8	211.0	169.4
Food wrapper	Paved	20	3.2	16	16	100.0	168.0	112.1	145.5
Food wrapper	Grass	0	2.3	16	7	43.8	10.2	7.8	8.0
Food wrapper	Grass	0	2.7	16	11	68.8	10.7	6.5	16.9
Food wrapper	Grass	0	3.2	16	13	81.3	23.9	14.2	26.1
Food wrapper	Grass	10	2.3	16	13	81.3	38.6	7.2	63.9
Food wrapper	Grass	10	2.7	16	13	81.3	43.7	1.4	64.2
Food wrapper	Grass	10	3.2	16	16	100.0	174.4	168.8	137.8
Food wrapper	Grass	20	2.3	16	12	75.0	7.4	1.1	8.7
Food wrapper	Grass	20	2.7	16	16	100.0	91.7	63.0	101.0
Food wrapper	Grass	20	3.2	16	16	100.0	118.9	39.5	145.7
Drinking cup	Paved	0	2.3	24	18	75.0	19.6	7.9	23.8
Drinking cup	Paved	0	2.7	24	21	87.5	17.2	8.5	22.2
Drinking cup	Paved	0	3.2	24	22	91.7	19.7	8.1	33.6
Drinking cup	Paved	10	2.3	16	16	100.0	165.0	39.2	195.1
Drinking cup	Paved	10	2.7	16	16	100.0	129.6	49.6	157.8
Drinking cup	Paved	10	3.2	16	16	100.0	236.8	81.9	262.0
Drinking cup	Paved	20	2.3	16	16	100.0	118.8	91.9	87.0
Drinking cup	Paved	20	2.7	16	16	100.0	147.0	90.9	118.9
Drinking cup	Paved	20	3.2	16	16	100.0	238.9	237.7	130.7
Drinking cup	Grass	0	2.3	24	10	41.7	1.1	0.4	1.5
Drinking cup	Grass	0	2.7	24	12	50.0	3.0	0.3	5.0
Drinking cup	Grass	0	3.2	24	17	70.8	16.5	10.7	23.9
Drinking cup	Grass	10	2.3	24	22	91.7	36.5	0.7	50.3

Drinking cup	Grass	10	2.7	24	22	91.7	55.0	17.2	67.5
Drinking cup	Grass	10	3.2	24	24	100.0	123.7	87.0	105.1
Drinking cup	Grass	20	2.3	24	19	79.2	66.1	32.8	88.4
Drinking cup	Grass	20	2.7	24	21	87.5	118.6	68.9	125.0
Drinking cup	Grass	20	3.2	24	24	100.0	175.4	173.1	136.7
Bag	Paved	0	2.3	16	16	100.0	350.0	366.6	160.1
Bag	Paved	0	2.7	16	16	100.0	318.8	310.0	185.1
Bag	Paved	0	3.2	16	16	100.0	612.2	573.0	188.2
Bag	Paved	10	2.3	16	16	100.0	433.7	424.8	131.8
Bag	Paved	10	2.7	16	16	100.0	436.5	428.8	116.3
Bag	Paved	10	3.2	16	16	100.0	493.1	502.6	186.8
Bag	Paved	20	2.3	16	16	100.0	266.9	284.8	127.9
Bag	Paved	20	2.7	16	16	100.0	415.0	397.9	176.7
Bag	Paved	20	3.2	16	16	100.0	460.2	462.3	143.0
Bag	Grass	0	2.3	16	16	100.0	111.1	18.0	167.0
Bag	Grass	0	2.7	16	15	93.8	146.6	191.7	106.4
Bag	Grass	0	3.2	16	16	100.0	217.9	202.3	136.9
Bag	Grass	10	2.3	16	16	100.0	175.5	166.1	96.9
Bag	Grass	10	2.7	16	15	93.8	213.5	241.0	156.8
Bag	Grass	10	3.2	16	16	100.0	290.9	316.5	154.7
Bag	Grass	20	2.3	16	16	100.0	204.5	198.4	176.3
Bag	Grass	20	2.7	16	16	100.0	304.4	303.4	132.9
Bag	Grass	20	3.2	16	16	100.0	433.1	416.4	213.8

Table S2. Results from the rain experiments. The mobilization probability, mean and median transport velocities (and standard deviation) are listed for all tested combinations of plastic item type, terrain type and slope angle. Transport velocities are expressed in meters per hour (m/h). Purely gravity driven transport of plastic items are excluded from the 'Total number of items tested'.

Type of plastic item	Terrain type	Terrain slope angle (°)	Rain intensity (mm/min)	Total number of items tested	Total number of moved items	Mobilization probability (%)	Mean velocity of moved items (m/h)	Median velocity of moved items (m/h)	STD (m/h)
Bottle	Paved	10	1.4	0	*n/a*	*n/a*	*n/a*	*n/a*	*n/a*
Bottle	Paved	10	2.0	16	12	75.0	0.2	0.2	0.2
Bottle	Paved	10	3.3	16	12	75.0	0.2	0.1	0.3
Bottle	Paved	20	1.4	16	14	87.5	102.5	7.4	119.3
Bottle	Paved	20	2.0	16	16	100.0	97.8	45.3	100.4
Bottle	Paved	20	3.3	16	16	100.0	113.0	18.5	125.2
Bottle	Grass	10	1.4	24	0	0.0	*n/a*	*n/a*	*n/a*
Bottle	Grass	10	2.0	24	0	0.0	*n/a*	*n/a*	*n/a*
Bottle	Grass	10	3.3	24	4	16.7	0.1	0.1	0.0
Bottle	Grass	20	1.4	24	1	4.2	0.1	0.1	0.0
Bottle	Grass	20	2.0	24	13	54.2	0.7	0.1	2.1
Bottle	Grass	20	3.3	24	21	87.5	11.0	0.3	30.5
Food wrapper	Paved	10	1.4	0	*n/a*	*n/a*	*n/a*	*n/a*	*n/a*
Food wrapper	Paved	10	2.0	16	7	43.8	0.2	0.1	0.2
Food wrapper	Paved	10	3.3	16	5	31.3	0.5	0.2	0.5
Food wrapper	Paved	20	1.4	0	*n/a*	*n/a*	*n/a*	*n/a*	*n/a*
Food wrapper	Paved	20	2.0	16	8	50.0	0.1	0.1	0.1
Food wrapper	Paved	20	3.3	16	11	68.8	0.3	0.2	0.6
Food wrapper	Grass	10	1.4	16	9	56.3	0.1	0.1	0.0
Food wrapper	Grass	10	2.0	16	14	87.5	0.1	0.1	0.0
Food wrapper	Grass	10	3.3	16	13	81.3	0.2	0.1	0.2
Food wrapper	Grass	20	1.4	16	13	81.3	0.1	0.1	0.1

Food wrapper	Grass	20	2.0	16	14	87.5	0.5	0.3	0.6
Food wrapper	Grass	20	3.3	16	15	93.8	0.9	0.5	1.0
Drinking cup	Paved	10	1.4	0	*n/a*	*n/a*	*n/a*	*n/a*	*n/a*
Drinking cup	Paved	10	2.0	16	13	81.3	8.1	4.9	9.0
Drinking cup	Paved	10	3.3	16	14	87.5	53.0	19.3	81.1
Drinking cup	Paved	20	1.4	16	16	100.0	81.6	16.7	121.3
Drinking cup	Paved	20	2.0	16	16	100.0	57.2	7.7	87.5
Drinking cup	Paved	20	3.3	8	8	100.0	2.0	2.1	0.7
Drinking cup	Grass	10	1.4	24	0	0.0	*n/a*	*n/a*	*n/a*
Drinking cup	Grass	10	2.0	24	1	4.2	0.1	0.1	0.0
Drinking cup	Grass	10	3.3	24	9	37.5	0.1	0.1	0.0
Drinking cup	Grass	20	1.4	24	6	25.0	22.2	0.1	49.5
Drinking cup	Grass	20	2.0	24	14	58.3	26.5	6.7	49.2
Drinking cup	Grass	20	3.3	24	22	91.7	50.2	0.2	96.7
Bag	Paved	10	1.4	0	*n/a*	*n/a*	*n/a*	*n/a*	*n/a*
Bag	Paved	10	2.0	16	14	87.5	0.3	0.3	0.1
Bag	Paved	10	3.3	16	12	75.0	0.3	0.3	0.1
Bag	Paved	20	1.4	0	*n/a*	*n/a*	*n/a*	*n/a*	*n/a*
Bag	Paved	20	2.0	16	8	50.0	0.3	0.1	0.4
Bag	Paved	20	3.3	16	8	50.0	0.3	0.1	0.3
Bag	Grass	10	1.4	16	11	68.8	0.1	0.1	0.0
Bag	Grass	10	2.0	16	11	68.8	0.1	0.1	0.0
Bag	Grass	10	3.3	16	14	87.5	0.2	0.1	0.1
Bag	Grass	20	1.4	16	13	81.3	0.1	0.1	0.1
Bag	Grass	20	2.0	16	15	93.8	0.2	0.1	0.1
Bag	Grass	20	3.3	16	14	87.5	0.2	0.2	0.1

Supplementary Figures

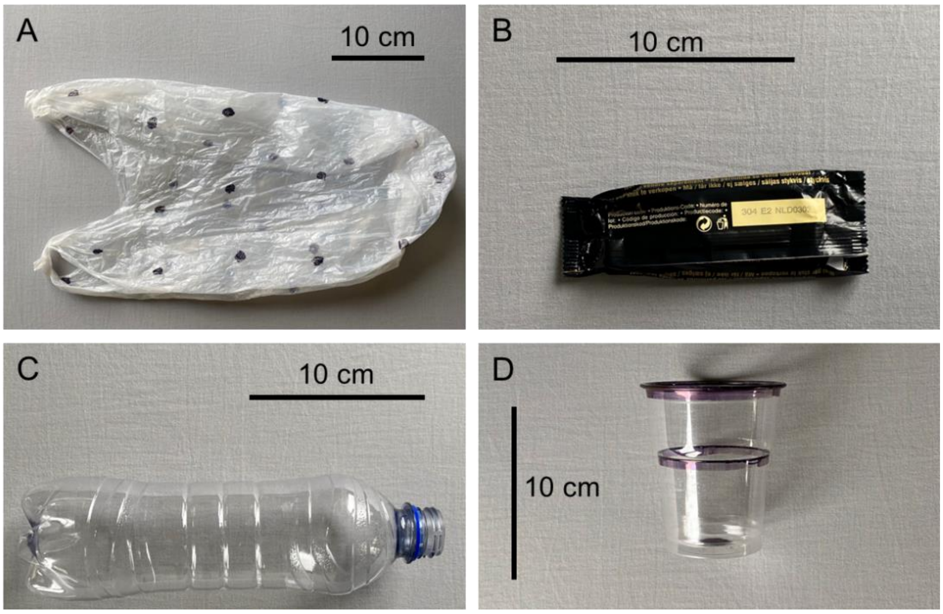

Figure S1. The four macroplastic items that were used in the experiments of this study: plastic bags (A), foodwrappers (B), water bottles (C), and drinking cups (D).

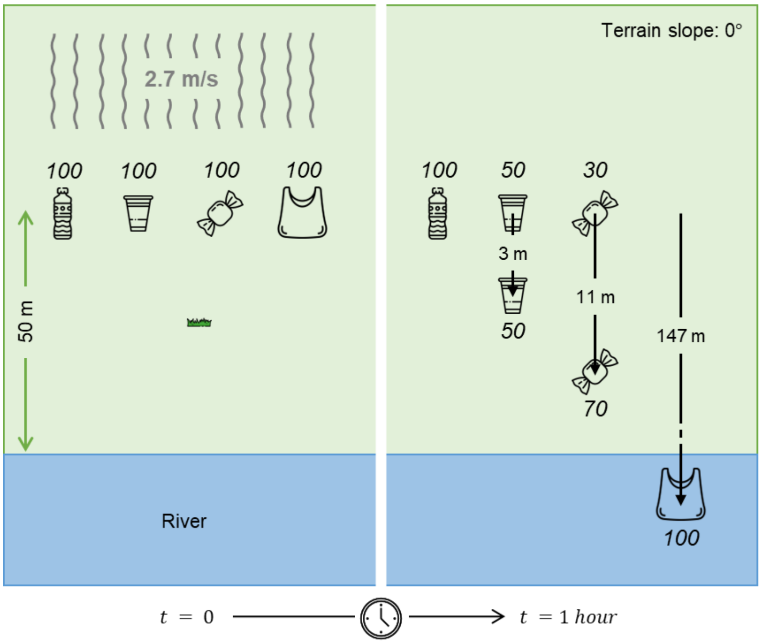

Figure S2. The concept of selective macroplastic mobilization and transport on land. Example for wind-driven macroplastic mobilization and transport. Schematic top view of a river channel and adjacent grass area (terrain slope angle of 0°) on which a mixture of bottles, cups, wrappers and bags (100 of each) is located. The plastic items are initially 50 meters away from the edge of the river channel. Hypothetically a wind speed of 2.7 m/s occurs for 1 hour. Based on the mobilization probabilities found in our experiments, this would mobilize 0% of the bottles, 50% of the cups, 30% of the food wrappers, and 100% of the bags. Based on the transport velocities found in our experiments, the cups, food wrappers and bags would have displaced 3, 10 and 147 meters, respectively, in one hour. In this case it would mean that all bags, which is 25% of the initial waste mixture, has reached the river.

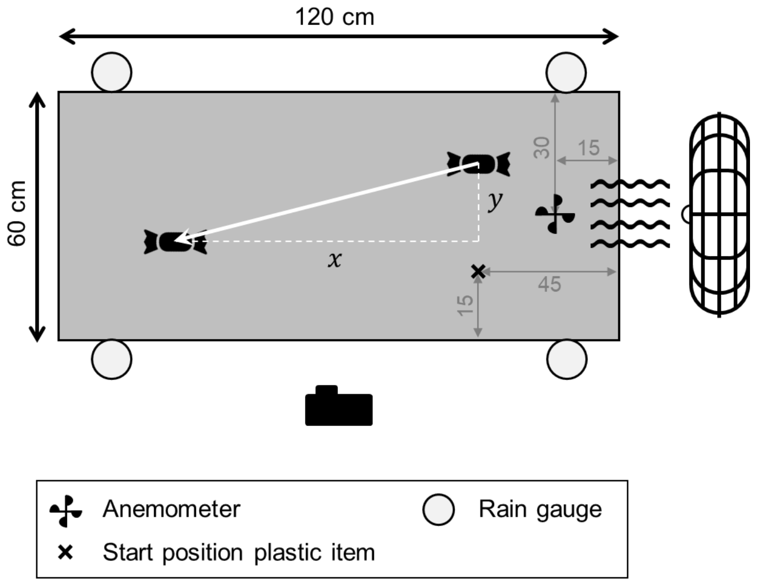

Figure S3. Schematic top view of the experimental set up. The artificial surface dimensionas are indicated along with the locations of the wind fan, the GoPro camera, the anemometer (only used in the wind experiments), the rai ngauses, and the start locations of the two plastic items. As an example one plastic item is shown with a hypothetical displacement vector and the corresponding $\mathbf{x}$ and $\mathbf{y}$ components. Distances are in centimeters.
